# Neighbourhood socio-economic disadvantage and loneliness: the contribution of green space quantity and quality

**DOI:** 10.1186/s12889-023-15433-0

**Published:** 2023-03-30

**Authors:** Tara Jamalishahni, Gavin Turrell, Sarah Foster, Melanie Davern, Karen Villanueva

**Affiliations:** 1 Centre for Urban Research, RMIT University, Melbourne, VIC 3000 Australia; 2 Policy and Equity, Murdoch Children’s Research Institute, Melbourne, VIC 3052 Australia; 3 School of Agriculture and Environment, The University of Western Australia, Perth, Australia; 4 Centre for Health Equity, Melbourne School of Global and Population Health, University of Melbourne, Victoria, 3010 Australia

**Keywords:** Neighbourhood disadvantage, Loneliness, Green space

## Abstract

Disadvantaged areas experience higher levels of loneliness than advantaged areas, though studies rarely identify environmental determinants of neighbourhood inequity in loneliness. We studied the contribution of the quantity and quality of green space to neighbourhood inequity in loneliness in three buffer sizes (400 m, 800 m, 1600 m), using cross-sectional data from 3778 individuals aged 48–77 years old living in 200 neighbourhoods in Brisbane, Australia. Levels of loneliness were significantly higher in disadvantaged neighbourhoods, and these neighbourhoods had less green space and less access to quality green space. However, there was no evidence that neighbourhood disparities in green space contributed to the association between neighbourhood disadvantage and loneliness. Possible methodological and substantive reasons for this result are discussed.

**Supplementary Information:**

The online version contains supplementary material available at 10.1186/s12889-023-15433-0.

## Introduction

Loneliness is emerging as an important health and wellbeing concern for individuals and society, with numerous negative health [1] and economic consequences [2]. Loneliness predicts increased mortality [3], high systolic blood pressure [4], coronary heart disease [5], and depression [6]. The cost of loneliness to the healthcare system should not be ignored - for example, in Australia, lonely people visit doctors more frequently than non-lonely people, and their hospital admission rate is nearly double that of those who are not lonely [2].

Loneliness can be defined as a feeling that originates from fewer social relationships [7] or from incongruent matching between perceived actual and desired social relationships [8]. While loneliness can be related to fewer social relationships, the most important predictor is a lack of quality social relationships [9]. Quality social relationships can protect all age groups from loneliness, but their importance might vary across age groups. As individuals age, the quality of social relationships becomes more critical than the quantity of relationships[10]. Psychosocial resources, such as place attachment, and social cohesion, can influence the quality of social relationships [145, 144, 143, 142]. Older adults who are involved in community activities have the potential to develop such resources [11], [12]. However, not all older adults have such opportunities, and due to several factors associated with aging and life stage conditions, older people are at a high risk of loneliness [13]. They are more likely to live alone, have lost peers and relatives, and experience mental and physical illnesses like dementia and weakness in physical function. More than 30% of adults aged over 45 years feel lonely in the United States, England, and Ireland [14–17]. This proportion is likely to increase with aging populations (over 65) and is predicted to double by 2050 [18]. Consequently, loneliness is expected to affect an increasing number of older adults in the future and is a critical social issue that requires further investigation.

Loneliness is not equitably distributed in society and has been found to be higher in disadvantaged neighbourhoods [19–23]. Health inequity encompasses the notion that disparities in health outcomes are caused by unjust policies and societal structures [24]. The neighbourhood inequity in loneliness might be related to disadvantaged populations having unequal access to resources such as employment, education, health care, public transport [25], and access to affordable health-promoting resources like green spaces [26], [27]. Therefore, inequality in access to green space can contribute to loneliness across neighbourhood disadvantages.

Green spaces are important health-promoting resources that can advance health equity [28]. The benefit of green space on health is the strongest for those with low individual-level socio-economic status and those residing in more disadvantaged neighbourhoods [29–31]. Generally, this might be because disadvantaged neighbourhoods are often home to people living with socio-economic disadvantages who rely on green spaces in their close proximity and are more likely to spend time around their homes [32]. Furthermore, most disadvantaged people may use green spaces more frequently due to a lack of other affordable recreation options [33–35].

Green spaces improve health and wellbeing through several mechanisms, such as providing nature and chance for social interaction [32]. Green spaces provide an opportunity for interaction with nature, which results in exposure to fresh air, the development of healthier behaviours (e.g., physical activity and better sleep), attention restoration and stress recovery [32]. Green spaces also provide spaces for socialisation and social wellbeing [36]. These spaces can foster social interaction by enabling residents to get social recognition and build ties with the community [37–40]. This is especially true for populations in disadvantaged neighbourhoods [41], [42]. The authors in [41] found that vegetation levels predict both the use of public spaces and the depth of neighbourhood social ties. Moreover, they showed that the use of public spaces mediated the association between vegetation and social ties in the neighbourhood. When neighbourhood social ties are strong, social support and social cohesion in neighbourhoods can enhance [43]; both social support and cohesion are important mediators of health inequity [44] and can help to alleviate loneliness [45]. With numerous identified social, health and environmental benefits [32], green spaces are increasingly being referred to as urban green infrastructure (UGI) [46], defined by the authors in [47] (p. 128) as a “network of planned and unplanned green spaces, spanning both the public and private realms, and managed as an integrated system to provide a range of benefits. Urban Green Infrastructure can include remnant vegetation, parks, private gardens, golf courses, street trees and more engineered options, such as green roofs, green walls, biofilters and rain gardens”. Green infrastructure is an important component of “social infrastructure” that describes social service needs across the lifespan that are generally government-funded [48].

There is great interest in the role of green space in health [49], [50] and health inequities, which have continued to grow in recent times [28], [51], [52], particularly with a focus on older adults [53–56]. However, most studies are about physical health inequities like cardiovascular health/disease, obesity, and general health [28], and some studies are about mental health inequities like neighbourhood inequity in depression and distress [51–54], [57], [58]. Little is known about the role of green space in loneliness and neighbourhood inequity in loneliness. Studies about the role of green space in loneliness have mainly focused on the influence of green space quantity (including distance and percentage of green space) in loneliness [30], [31], [59], [60], and few have explored the influence of quality of green space on loneliness and the influence of quantity and quality of green space on neighbourhood inequity in loneliness [59], [61].

The results of studies about the impact of the quantity of green space on loneliness are inconsistent. Two studies found that time spent in green space [62], and the percentage of green space were negatively associated with loneliness [30], while others found no or marginal associations [31], [59–61], [63]. Although these inconsistencies may arise from different measures and analysis methods, it also suggests the need for further investigation in different neighbourhood contexts. Moreover, none of these studies considered the quality of green space. Green spaces with poor quality may discourage residents from using them [64], [65]. Authors in [66] state that green spaces enable individuals to socialise, see and hear their neighbours. Investigating the use of green space, the authors in [66] found that spending time in public green spaces can reduce loneliness and increase neighbourhood social cohesion. The critical role of quality green spaces has also been highlighted during COVID lockdowns, with green spaces overflowing with picnicking families, children playing, dog walkers, and cyclists [67]. Accessible and good-quality green spaces that include facilities (e.g., easy access to a toilet, accessible pathways, safety, green space maintenance, availability of grass and trees and seating areas) are preferred and more likely to be used by older adults [68–74].

This study aims to improve understanding of the potential influence of quantity and quality of green space on neighbourhood inequity in loneliness across different levels of neighbourhood disadvantage in the middle to the older adult population. In this study, four hypotheses were examined. The first two hypotheses must be tested prior to testing the main third and final hypothesis. First, that loneliness would be higher in more disadvantaged neighbourhoods. Second, that the quantity and quality of green spaces would not be equally distributed across a city; greater quantity and quality of green space would more likely be found in advantaged than in disadvantaged neighbourhoods. Third, that quantity and quality of green space would be negatively associated with loneliness. Finally, that less access to quantity and quality of green space in disadvantaged neighbourhoods than in advantaged neighbourhoods would explain neighbourhood inequities in loneliness.

## Method

### Study population

This investigation uses data from the fifth wave (2016) of the HABITAT (How Areas in Brisbane Influence HealTh and AcTivity) study [75]. HABITAT is a multilevel longitudinal study of mid-aged adults living in the Brisbane Local Government Area (LGA) in Queensland, Australia. The Brisbane LGA is a medium-density jurisdiction with a population of 1.2 million people in 2016 [76], and the area is managed by a single city council or municipality [77]. The primary aim of HABITAT is to examine patterns of change in health and wellbeing over the period 2007–2016, and to assess the relative contributions of environmental, social, psychological, and sociodemographic factors. The HABITAT study received ethical clearance from the Queensland University of Technology Human Research Ethics Committee (Ref. Nos. 3967 H & 1300000161) and RMIT University (2022-25157-17608).

### Sample

Details regarding the baseline sampling of HABITAT have been published elsewhere [78], with only one person per household selected in the sample. Briefly, a multi-stage probability sampling design was used to select a stratified random sample (n = 200) of Census Collection Districts (CCD), and a random sample of people aged 40–65 years living in these CCD (n = 16,127, on average 85 persons per CCD). CCDs are embedded within a larger suburb, and the area corresponding to and immediately surrounding a CCD is likely to have meaning and significance for their residents. For this reason, we hereafter use the term ‘neighbourhood’ to refer to CCDs. The HABITAT (2007) baseline sample was broadly representative of the population aged 40–65 years living in the Brisbane LGA [79].

### Data Collection and Response Rates

A structured self-administered questionnaire was sent to 17,000 potentially eligible participants in May 2007 using a mail survey method developed by the author [80]. After excluding 873 out-of-scope contacts (i.e., deceased, no longer at the address, unable to participate for health-related reasons), 11,035 usable surveys were returned, yielding a baseline response rate of 68.3%. The corresponding response rates from in-scope and contactable participants in 2009, 2011, 2013, and 2016 were 72.6% (n = 7,866), 67.3% (n = 6,900), 67.1% (n = 6,520), and 58.7% (n = 5,187), respectively. This study used data from wave 5 with a sample size of 5,187.

### Neighbourhood-level measures

#### Neighbourhood socio-economic disadvantage

A socio-economic score was given to each of the 200 neighbourhoods using the Australian Bureau of Statistics Index of Relative Socio-economic Disadvantage (IRSD) [81]. The IRSD score for a neighbourhood, calculated using census data from 2016, represents the overall level of disadvantage in each neighbourhood as determined by variables that encompass a variety of socio-economic characteristics, such as education, occupation, income, unemployment, household structure, and tenure (among others). Based on their IRSD scores, the HABITAT neighbourhoods were classified into quintiles, with Q1 designating the 20% (n = 40 out of 200) of the most disadvantaged districts compared to all of Brisbane and Q5 designating the 20% with the greatest advantage. (n = 40 out of 200).

#### Green space

##### *Green space data sources*

The database used for measuring green space quantity was created by compiling data from five different databases (see Table 1): Queensland Cadastre Dataset [82]; Queensland Protected Areas [83], Queensland Recreation Areas [83], Brisbane City Council Park [86], and the Australian Urban Observatory [85]. These data sources include different types of green space (see Table 2): Reserve Parks, Conservation Parks, National Parks, Sport Parks, Recreation Parks, Corridor Link Parks, Natural Areas, Park Areas, Community Use Parks, Landscape Amenity Parks, Utility Parks, Unclassified Parks. Measures of green space quality were sourced from the Brisbane City Council Park database [86].

**Table 1:** Land use categories and data sources

Dataset	Land use categories included as green space	Data source
Queensland Cadastre Dataset	Reserve	[82]
Queensland Protected Areas	Conservation Park; National Park	[83]
Queensland Recreation Areas	Gardens; Golf Course; Miscellaneous Area. Oval Area; Racecourse; Racetrack. Recreation Area; Show Ground; Zoo	[83]
Brisbane City Council Park	Community Use Park; Corridor Link Park; Informal Use Park; Landscape Amenity Park; Natural Area Park; Sport Park; Unclassified parks	[86]
Australian Urban Observatory		[85]

**Table 2:** Descriptions of land use categories*

Name	Description
Reserve Parks [82]	“Over 27,000 parcels of land throughout Queensland have been set aside under the Land Act 1994 for a particular public or community purpose. These are either reserves or deeds of grant in trust and are collectively referred to as ‘trust land’. Many recreation facilities and parks and gardens, such as Anzac Park in Brisbane, are on trust land.”
Conservation Park and National Park [83]	Conservation Park and National Park are considered as protected areas. Protected areas of Queensland represent those areas protected for the conservation of natural and cultural values and those areas managed for the production of forest resources, including timber and quarry material.
Queensland Recreation Areas [87]	A general purpose or large park usually in a residential area.
Sport Park; Recreation Park; Corridor Link Park; Natural Area Park area; Community Use Park; Landscape Amenity Park; Utility Park; Unclassified parks [84].	*Sport Park* Park that provides an outdoor setting for formal, structured sports activities, including training, skills development and competition. Sport Park can be developed and used for one or more sport and is usually managed by a community sports club under a lease or license agreement with Council. *Recreation Park* Park provides an outdoor setting for recreation and social activities and events, including formal or structured activities and events (e.g., weddings, Parkrun) and informal or unstructured activities (e.g., picnics, walking). Recreation Park is usually available for public use at all times and can be used for a range of private and/ or commercial activities and events with Council consent. *Corridor Link Park* Park that comprises linear open, green spaces, such as creek corridors and road reserves, and is developed and managed to enable pedestrian and cyclist access and/ or protect and enhance important riparian and dryland corridor habitat. *Natural Area Park area* Park comprises areas of significant natural value, such as remnant bushland, koala habitat and protected vegetation, and is managed to protect and enhance these values as well as provide opportunities for the community to experience and learn about nature and natural values and participate in outdoor nature-based recreation. *Community Park* Park that provides a setting for community facilities and services, such as libraries, senior citizens centres, swimming pools, YMCAs, meals on wheels and guide/ scout huts. Community Park typically comprises a building or built facility with support infrastructure such as car parking. It can be single or multipurpose and is managed either by Council (e.g., library) or by a community or other organisation under a lease or license agreement with Council (e.g., aquatic centre, lapidary club). Public access to community park varies depending on the facility. *Landscape Amenity Park* Park that comprises areas of significant landscape and scenic amenity value, such as landmarks, signature points (e.g., a stand of significant trees), special landscape and natural features (e.g., a lake, rocky outcrop), views/ vistas and visual buffers (e.g., to incompatible land uses). Landscape amenity park is managed to protect and enhance these values and generally provides limited or no opportunities for recreational or community use. *Utility Park* Park that is developed and used for a range of utilities and services such as Council works depots, water reservoirs, quarries, high voltage power lines and roads. Utility Park is generally unrelated to other park functions and values and is not available (or suitable) for publicly access or use. *Unclassified Park* New Park has been added to the park database and is awaiting classification.
Australian Urban Observatory [85]	Public Open Space measures in the AUO include publicly available, parks and gardens, recreations fields, conservation areas (national parks, state forests, etc.), urban waterways (rivers, lakes, beaches etc.) and civic squares and promenades.

##### *Green space definition*

This study focused on publicly accessible green spaces. Green spaces include public gardens, parks, waterways, rivers, lakes, wetlands, conservation areas, beaches, some civic squares and other areas with grass, trees, and/or shrubs where people gather for leisure, social activities, and recreational purposes. Green spaces can be a mix of both soft (permeable) surfaces and hard (i.e., impervious) surfaces. Private residential gardens and private school grounds were not included because they are often fenced, and public access is not always permitted. Each public open space was checked, using aerial imagery (Google Earth), to ensure only public open spaces with green areas were included. Access to green spaces was based on objective spatial residential proximity to quantity (percentage of green spaces) and quality of green spaces.

##### *Buffer distances*

Using Esri’s ArcGIS 10.3 software (ESRI, Redlands, CA), green space measures were calculated within the Euclidean buffers of 400, 800 and 1600 m around the participant’s residential address. Different buffer sizes were selected to examine whether the quantity and quality of green space near a participant’s home has a stronger or weaker association with loneliness than those further away.

Buffer sizes of 400 and 800 m were chosen based on previous studies. The authors in [88] report that the median value of the buffer distances used in mental health studies is 400 m [88]. Also, a buffer size of 400 m, 5 min walking distance, is commonly used in urban planning and transportation research [89], [90]. An 800 m buffer is typically used to depict a “20-minute” return walk from home where local shops, parks, and a variety of amenities and facilities are accessible [91].

A 1600 m buffer size was also chosen to account for the range of walkable distance for older adults. For example, the author in [92] reported that the distance walked by older adults (+ 60 years) is between 700 and 1600 m, and a recent study found that older mid-aged adults (aged 50–64 years) walked between 600 and 2000 m overall to utilitarian destinations [93].

##### *Green space measures*

All spatial datasets were combined into a geographic information system (GIS) (ArcMap, ESRI version 10.3), and two objective green space measures were provided for each buffer size (400, 800 and 1600 m buffers): (1) the total percentage of green space; and: (2) mean the quality of green space.

##### *Quantity of green space*

The total percentage of areas of green space: the area of green space within each buffer was divided by the area of the buffer size.

##### *Calculating the percentage of green space*

Each green space dataset layer in Table 1 was cleaned by extracting green space types of interest (Table 1) and removing schools and private residential gardens. Clean layers were merged to form one unique layer. Using the analysis tool in GIS, the green space polygon layer intersected with the buffer polygon layer to identify the number of green spaces and the area of each green space within each buffer. Summary statistics were calculated to determine the total number and area of green spaces present within each buffer. The distribution of the percentage of green space in each buffer was skewed, so categories were created consistent with [94] as follows: <10%; >=10 and < 20%; >=20% and < = 30%; and > 30%).

##### *Quality of Green space*

Previous studies have used a mix of subjective and objective methods to measure the quality of green space, including the perceived quality of urban green space [95–97], auditing tools to capture park features and amenities^1^ as a proxy for quality [68], [70], [98–103], and composite quality scores (for the availability of some features like shade, walking and biking path, maintenance, and tennis court in green spaces)[104]. Our study used a composite measure by extracting objective data from an available dataset to minimise information loss [102], [105].

##### *Criteria for inclusion of facilities in the green space quality measure*

The inclusion of facilities was based on the following criteria: (1) age-friendliness, which includes features preferred by the older population (e.g. benches, toilets) [70], [72], [106]; (2) features that facilitate social activities (e.g., benches, picnic table, off-leash dog areas) [45], [107]; (3) features previously associated with park use in the HABITAT sample (e.g., BBQ, drinking fountains, lighting, public toilets, and direction Sign) [108]; and finally the availability of car parks, bike racks, and walking and biking paths, which provide infrastructure for different modes of transport to enhance access to green space [70].

##### *Facility items*

Based on the criteria above, we included 24 facilities: 1-Bike racks, 2-Carparks, 3-Natural features (like bird hide and urban forest), 4-Restaurants, kiosks or/and Shops, 5-Garden, 6-Blue spaces (e.g., fountain, river), 7-Shade (e.g. shade structure and shade sail), 8-Direction sign, 9-Picnic table, 10-Seat, 11-Rubbish bin, 12-Toilet, 13-Lighting, 14-Drinking fountain, 15-BBQ, 16-Dogs off-leash area, 17-Playground, 18-Informal sport (e.g., Basketball half courts, and BMX tracks) 19-Dog exercise areas, 20-Formal sport (e.g., Sports clubhouse, formally marked courts and fields), 21-Walking path, 22-Biking path, 23-Handrail fence, and 24-Maintenance (e.g., Gardens beds, and irrigation systems) [45], [70], [72], [106–108].

##### *Calculating the quality of green space*

To form the Green space features dataset for this study, the 24 facilities were extracted from the Brisbane City Council dataset [86], which provides the coordinates of each feature as a location defined by a point in a GIS. Summary statistics tools in GIS were used to calculate the total number of facilities available in each green space.

To calculate the green space quality score, the total number of facilities in each green space (range from 0 to 24) was divided by the green space area size (which was available in the green space feature dataset). To calculate the green space quality score of a buffer (a buffer includes one or more green spaces), we attributed the green space quality scores to the associated buffers by the join tool in GIS. Then, the table file was exported to SPSS software version 28.0.0.0 (IBM Crop., 2022). Using SPSS, the green spaces quality of each buffer was calculated by summing up the green spaces’ quality scores within a buffer and dividing by the size of green spaces’ area. This measure was categorised into two levels of higher and lower quality. The cut-off points for categorisation to higher and lower were based on the median of quality green space for each buffer size.

### Individual-level measures, covariates, and controls

#### Education

Respondents were asked to provide information about the greatest degree of education they had attained. Four categories were used to code responses: 1 = bachelor’s degree or higher (included postgraduate diploma, master’s degree, or doctorate), 2 = Diploma (associate or undergraduate), 3 = Certificate (trade or business certificate or apprenticeship), or 4 = no post-school qualifications.

#### Occupation

Respondents who were working at the time of the survey were asked to provide their work titles before listing their primary responsibilities. The Australian and New Zealand Standard Classification of Occupations was used to code this information (ANZSCO) [81]. The initial ANZSCO categorisation was reclassified into four groups for analysis: 1 = Professionals, 2 = White-collar employees, and 3 = Blue-collar employees, 4 = retired and not in the workforce.

#### Household income

Respondents were asked to apply a 14-category measure that was afterwards transcoded into six groups for analysis to show their annual total household income (including pensions, allowances, and investments): Less than 1 = AU$31,200, 2 = AU$31,200 − 51,999, 3 = AU$52,000–72,799, 4 = AU$72,800 − 93,599, 5 = AU$93,600 − 129,999, and 6 = More than AU$130,000, 7 = Not classified (i.e., indicated ‘Don’t know’ or ‘Don’t want to answer this’, or left the income question blank).

#### Age and gender

Respondents self-reported their date of birth and gender. The mean age for this sample was 61 years (range 48–77 years). The age variable was categorised into five groups: 1 = 45–55 years, 2 = 56–60 years, 3 = 61–67 years and 4 = 68 years and older. The proportion of men and women in this sample was 42% and 58%, respectively. Age was categorised to be consistent with the previous HABITAT studies [109], [110]. We conducted sensitivity analyses to check if the age variable’s categorisation impacted our results, which it didn’t, so we retained the categorised age measure. The cut-off points for the categorised age variable were based on having almost an equal number of respondents in each category.

#### Distance from Central District Business (CBD)

This measure was used in some modelling to adjust for spatial confounding. Our sub analysis also revealed that distance from the CBD confounded the relationship between neighbourhood disadvantage, loneliness, and green space. Distance from the CBD was available in HABITAT data set and obtained from the Geographical Information Systems (GIS) data by measuring the straight-line distance (km) between the CBD and each respondent’s dwelling.

### Loneliness

Loneliness was measured using a 3-item scale adapted from [111]. The stem-question and items asked: How often do you feel left out; isolated from others; or lack companionship? For each item, participants were asked to indicate 1 = hardly ever, 2 = some of the time, and 3 = often. The scale was derived by summing the three items and so had a range of 3–9 (mean 4.1, SD 1.5, median 3.0). The Cronbach alpha for the scale was 0.86. and the loneliness mean is comparable to other studies [112], [113].

### Statistical analysis

For analysis, people who completed a survey on behalf of a sampled HABITAT participant were excluded (n = 93). This sometimes occurred when a sampled participant asked a relative or partner to complete the survey on their behalf, or when the partner of a deceased participant completed the survey. As these persons are not a member of the original HABITAT cohort they were excluded.

Moreover, some participants were excluded because they moved to a different neighbourhood after 2007 and were no longer living in one of the original 200 HABITAT neighbourhoods (n = 1274). Participants with missing data for loneliness (n = 34) and education (n = 8) were also excluded. These exclusions reduced the analytic sample to 3,778. Sensitivity analyses (not presented here) showed that those excluded due to missing data did not differ significantly from included participants on education, neighbourhood disadvantage or loneliness. To check multi-collinearity among variables, we calculated variance inflation factors (VIFs), with a value above 2.5 indicating a high level of multi-collinearity. All variables had a VIF below 2.5 [114].

Examining the contribution of green space quantity and quality to inequity in loneliness was based on a four-step approach using multilevel linear regression analysis, where individual measures were added at level one and neighbourhood measures at level two. Prior to evaluating the primary third, and final hypotheses, it is necessary to examine the first two analysis steps.

Step 1: To address the first hypothesis, loneliness (the dependent variable) was regressed on neighbourhood disadvantage (the independent variable) with adjustment for age and sex, and then with further adjustment for education, occupation, income and distance to CBD using MLwiN software version 28 [115].

Step 2: To address the second hypothesis, the quantity and quality of green space scores within 400, 800, and 1600 m of each participant’s home by different levels of neighbourhood disadvantage was examined by cross-tabulation in the SPSS software version 28.0.0.0 (IBM Crop., 2022).

Step 3: To address the third hypothesis, loneliness was regressed on each green space measure with adjustment for all the above-mentioned covariates.

Step 4: To address the last hypothesis, loneliness was regressed on neighbourhood disadvantage (model one), and then each of the six green space measures was separately added (models 2 to 7).

Interaction analyses were also conducted to test whether the association between neighbourhood disadvantage and loneliness varied depending on different levels of green space quantity and quality.

## Results

### Individual and neighbourhood-level measures and loneliness

In Table 3, the incidence of loneliness is shown by individual and area-level factors. People in the most disadvantaged neighbourhoods had the highest mean loneliness scores at the area level. Those with the lowest levels of education, those from low-income households, those in blue-collar occupations, and men scored the most lonely on average (although the latter was nonsignificant) at the individual level. Although not statistically significant, mean loneliness scores were highest in buffers with the lowest percentage of green space (10–20%) in the 400 and 1600 m buffer sizes, and higher in all buffers with higher quality green space.

**Table 3:** Neighbourhood disadvantage, green space measures, and sociodemographic characteristics of the analytic sample by mean loneliness (95% confidence interval)

	N (%)	Mean Loneliness	95% CI
**Neighbourhood disadvantage** ^**1**^			
Q1(most disadvantaged)	513 (13.5)	4.36	4.22, 4.51
Q2	569 (15.0)	4.23	4.10, 4.36
Q3	753 (19.9)	4.19	4.07, 4.31
Q4	852 (22.5)	3.98	3.88, 4.07
Q5(least disadvantaged)	1091 (28.8)	3.91	3.82, 3.99
P-value^2^		< 0.001	
**Age (years)**			
45–55	1020 (27.0)	4.21	4.11, 4.31
56–60	809 (21.4)	4.14	4.04, 4.25
61–67	1052 (27.8)	4.07	3.98, 4.16
68+	897 (23.7)	3.92	3.83, 4.01
P-value		< 0.001	
**Sex**			
Male	1585 (41.9)	4.11	4.04, 4.19
Female	2193 (58.0)	4.07	4.01, 4.13
		0.209	
**Education**			
No-post-school qualifications	1420 (37.5)	3.99	3.91, 4.06
Certificate	440 (11.6)	4.12	3.97, 4.26
Diploma	642 (16.9)	4.14	4.02, 4.25
Bachelor’s degree or higher	1276 (33.7)	4.17	4.08, 4.25
P-value		< 0.01	
**Household Income (AUD)**			
Less than 31,200	542 (14.3)	4.49	4.35, 4.64
31,200 − 51,999	560 (14.8)	4.19	4.06, 4.32
52,000–72,799	470 (12.4)	4.08	3.94, 4.22
72,800 − 93,599	422 (11.1)	4.08	3.95, 4.20
93,600 − 129,999	475 (12.5)	3.78	3.69, 3.88
+ 130,000	755 (19.9)	4.03	3.91, 4.16
Unclassified	554 (14.6)	4.49	4.35, 4.64
P-value		< 0.001	
**Occupation**			
Professional	1137 (30.1)	3.91	3.84, 3.99
White-Collar	594 (15.7)	4.25	4.11, 4.38
Blue-Collar	322 (8.5)	4.27	4.10, 4.44
Not in workforce	1725 (45.7)	4.11	4.04, 4.18
P-value		< 0.001	
**Percentage of green space 400 m buffer size**			
< 10%	1620 (42.9%)	4.04	3.97, 4.11
10–20%	912 (24.1%)	4.18	4.08, 4.28
20–30%	642 (17.0%)	4.04	3.92, 4.15
> 30%	604 (16.0%)	4.14	4.01, 4.26
P-value		0.088	
**Percentage of green space 800 m buffer size**			
< 10%	764 (20.2%)	4.10	3.99, 4.20
10–20%	1410 (37.3%)	4.08	4.01, 4.16
20–30%	859 (22.7%)	4.10	4.00, 4.20
> 30%	745 (19.7%)	4.08	3.97, 4.18
P-value		0.983	
**Percentage of green space 1600 m buffer size**			
< 10%	215 (5.7%)	4.04	3.83, 4.25
10–20%	1660 (43.9%)	4.12	4.05, 4.20
20–30%	1159 (30.7%)	4.06	3.97, 4.14
> 30%	744 (19.7%)	4.08	3.97, 4.18
P-value		0.650	
**Quality of green space 400 m buffer size**			
Lower quality	1845 (48.8%)	4.05	3.98, 4.12
Higher quality	1933 (51.2%)	4.12	4.06, 4.19
P-value		0.358	
**Quality of green space 800 m buffer size**			
Lower quality	1950 (51.6%)	4.07	4.01, 4.14
Higher quality	1828 (48.4%)	4.10	4.03, 4.17
P-value		0.082	
**Quality of green space 1600 m buffer size**			
Lower quality	1970 (52.1%)	4.05	3.98, 4.11
Higher quality	1808 (47.9%)	4.13	4.06, 4.20
P-Value		0.552	

1 Q1 to Q5 are quintiles

2 P-value derived from Welch test

### Neighbourhood disadvantage and loneliness

**Fig. 1:**
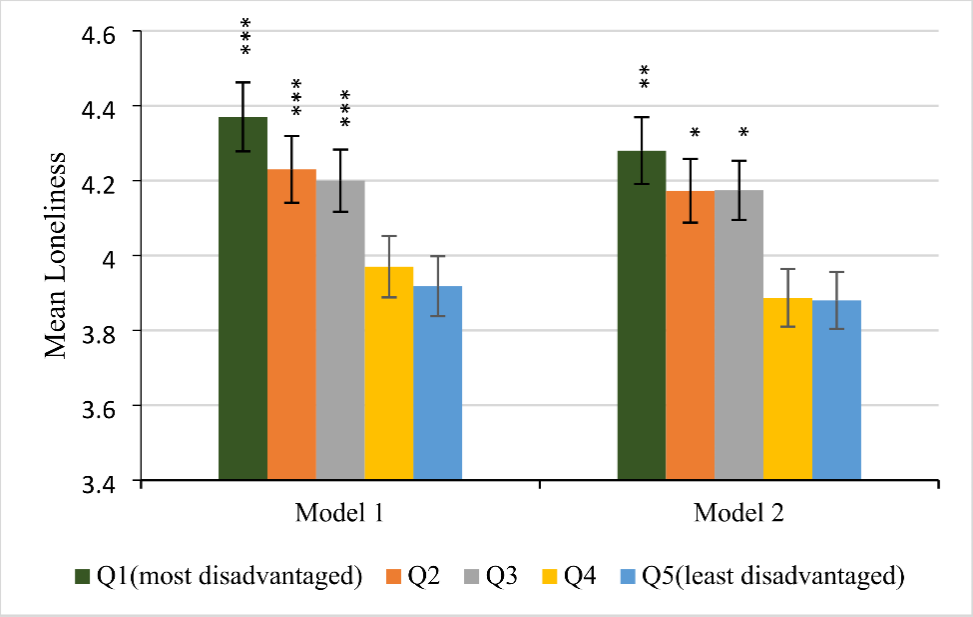
Association between neighbourhood disadvantage and loneliness. Model 1: adjusted for age and sex. Model 2: Model 1 plus adjustments for education, occupation, household income, and distance from CBD. Quintile 5 (Q5) represents the most advantaged neighbourhoods and is also the reference group. * p < 0.05. ** p < 0.01. *** p < 0.001

In terms of the first hypothesis, we discovered that, after controlling for age and sex, there was a hierarchical association between neighbourhood disadvantage and loneliness (Fig. 1, Model 1). Comparing residents of advantaged and disadvantaged neighbourhoods, loneliness was found to be much more prevalent in the latter group. The study found that there was a difference in reported loneliness between residents of disadvantaged and advantaged neighbourhoods. However, this difference was reduced by 2.6% in most disadvantaged neighbourhoods, 1.8% in highly disadvantaged neighbourhoods, and 1.1% in medium disadvantaged neighbourhoods after considering education, occupation, household income, and distance from the central business district. Despite these adjustments, the difference in loneliness between the two groups remained considerably significant. (Model 2).

### Green space and neighbourhood disadvantage

In terms of the second hypothesis, we presented the results of the cross-tabulation of green space variables for each buffer size and each neighbourhood disadvantage quartile (Tables 4 and 5). The total green space area and quality of green space were greater for participants living in the least disadvantaged areas.

**Table 4:** Quality of green space scores within 400 m, 800 m, and 1600 m of participant’s home by different levels of neighbourhood disadvantage

Neighbourhood disadvantage	Quality of green space 400 m buffer size		Quality of green space 800 m buffer size		Quality of green space 1600 m buffer size	
	Lower Quality %	Higher quality %	Lower Quality %	Higher quality %	Lower Quality %	Higher quality %
Q1(most disadvantaged)	58.1	41.9	66.5	33.5	56.9	43.1
Q2	43.1	56.9	51.5	48.5	59.8	40.2
Q3	47.9	52.1	49.5	50.5	45.4	54.6
Q4	43.8	56.2	43.4	56.6	47.3	52.7
Q5(least disadvantaged)	50.2	49.8	54.4	45.6	52.5	47.5
P-value*	< 0.001		< 0.001		< 0.001	

*P-value based on a Pearson Chi-Square test

**Table 5:** Percentage of green space within 400 m, 800 m, and 1600 m of participant’s home by neighbourhood disadvantage

Neighbourhood disadvantage	Percentage of green space in 400 m buffer size				Percentage of green space in 800 m buffer size				Percentage of green space in 1600 m buffer size			
	< 10%	10–20%	20–30%	> 30%	< 10%	10–20%	20–30%	> 30%	< 10%	10–20%	20–30%	> 30%
Q1(most disadvantaged)	51.7	23.6	13.6	11.1	24.4	49.3	18.5	7.8	12.5	67.4	12.3	7.8
Q2	50.8	21.4	16	11.8	16.7	44.5	23.4	15.5	5.6	39.5	35.5	19.3
Q3	44.6	28.3	13.9	13.1	20.2	49.3	17.5	13	3.6	49.3	37.3	9.8
Q4	44	21.9	14.7	19.4	24.9	25.4	21.5	28.3	6	40	34.5	19.5
Q5(least disadvantaged)	32.5	24.7	23	19.8	16.5	29.1	29	25.5	3.8	34.6	29.2	32.4
P-value*	< 0.001				< 0.001				< 0.001			

* P-value based on a Pearson Chi-Square test

### Green space quantity and quality factors and loneliness

In terms of the third hypothesis, we examined the association between loneliness and access to green space quantity and quality in different buffer sizes, adjusting for all covariates (Table 6). There was no significant association between the quantity and quality of green space and loneliness in any buffers, irrespective of their size.

**Table 6:** Associations between the green space measures and loneliness

Built environment	Model^1^	
	β	95% CI
**Percentage of green space 400 m buffer size**		
< 10%	Ref	
10–20%	0.073	-0.01, 0.16
20–30%	-0.005	-0.10, 0.09
> 30%	0.078	-0.02, 0.18
**Percentage of green space 800 m buffer size**		
< 10%	Ref	
10–20%	-0.023	-0.12, 0.07
20–30%	0	-0.10, 0.10
> 30%	-0.009	-0.12, 0.10
**Percentage of green space 1600 m buffer size**		
< 10%	Ref	
10–20%	0.049	-0.10, 0.20
20–30%	0.026	-0.13, 0.18
> 30%	0.063	-0.10, 0.23
**Quality of green space 800 m buffer size** Lower Higher		
	0.037	-0.03, 0.10
**Quality of green space 800 m buffer size** Lower Higher	Ref	
	0.061	-0.01, 0.13
**Quality of green space 1600 m buffer size** Lower Higher	Ref	
	0.035	-0.04, 0.11

1.Model: adjusted for age and sex, education, occupation, household income, and distance from CBD

### Neighbourhood disadvantage and loneliness adjusting for green space quantity and quality factors

In terms of the final hypothesis, seven models are presented in Table 7. Model 1, as baseline analysis, shows an association between neighbourhood disadvantage and loneliness adjusted for age, sex, education, occupation, household income, and distance from CBD. Models 2 to 7 expand on Model 1 with adjustments for the percentage of green space within 400 m, 800 m, and 1600 m and the quality of green space within 400 m, 600 m, and 800 m. There was no attenuation in the positive baseline association between neighbourhood disadvantage and loneliness (Model 1) after adjustment for green space quantity (Models 2 to 4) and quality (Models 5 to 7) within the different buffer distances.

We tested the interaction between each measure of quantity and quality of green space and loneliness by neighbourhood disadvantage. We ran six interaction models to see if the association between neighbourhood disadvantage and loneliness varied by different levels of access to green space quantity and quality. No significant interactions were found (results are provided as supplementary material).

**Table 7:** Modelling the association between neighbourhood disadvantage and loneliness, adjusting for the covariates^1^

Neighbourhood disadvantage (quintiles)					
	Q5 Least disadvantaged	Q4	Q3	Q2	Q1 Most disadvantaged
Model 1	Ref	0.013, (-0.09, 0.11)	0.131, (0.03, 0.23)*	0.137, (0.02, 0.25)*	0.211, (0.09, 0.33)**
Model 2	Ref	0.019, (-0.08, 0.12)	0.138, (0.04, 0.24)*	0.155, (0.04, 0.27)*	0.227, (0.11, 0.35)***
Model 3	Ref	0.012, (-0.09, 0.11)	0.144, (0.04, 0.25)*	0.147, (0.03, 0.26)*	0.225, (0.10, 0.35)***
Model 4	Ref	0.023, (-0.08, 0.12)	0.146, (0.04, 0.25)*	0.149, (0.03, 0.26)*	0.233, (0.11, 0.36)***
Model 5	Ref	0.011, (-0.09, 0.11)	0.130, (0.03, 0.23)*	0.133, (0.02, 0.25)*	0.212, (0.09, 0.33)***
Model 6	Ref	0.005, (-0.09, 0.10)	0.127, (0.03, 0.23)*	0.132, (0.02, 0.25)*	0.215, (0.10, 0.33)***
Model 7	Ref	0.010, (-0.09, 0.11)	0.128, (0.03, 0.23)*	0.135, (0.02, 0.25)*	0.207, (0.09, 0.33)**

1. Linear regression coefficients and their 95% confidence intervals.

## Discussion

This research aimed to improve understanding of the potential influence of green space quantity and quality on loneliness and neighbourhood inequity in loneliness. Results of our study showed that residents of socioeconomically disadvantaged neighbourhoods were significantly more likely to be lonely than residents of advantaged neighbourhoods, which is consistent with the findings of previous research [19], [21–23], [109], [116]. To understand why loneliness is higher in disadvantaged neighbourhoods, we examined the contribution of differences in access to quantity and quality of green space between disadvantaged and advantaged neighbourhoods.

We first hypothesised that access to quantity and quality of green space differed between disadvantaged and advantaged neighbourhoods. Our results showed that the quality of green space is poorer, and the quantity is smaller in disadvantaged compared to advantaged neighbourhoods, which concurs with other studies [27][27][51], [53], [56], [117–119]. We also hypothesised that higher quality and greater amount of green space would be associated with lower levels of loneliness, but we observed no association to support this. Neither access to the quality of green space nor the percentage of green space in different buffer sizes was associated with loneliness. Our study is consistent with some findings [59], [61] and contradicts another [120]. In the Australian context, a four-year longitudinal study conducted by authors in [59] found that access to green space in 400 and 800 m buffers of residents’ homes had no impact on loneliness, but green space in 1600 m buffers was marginally negatively associated with loneliness among working-aged adults. Specifically, residents with access to more than 30% green space within a 1600 m Euclidean buffer experienced a significantly lower likelihood of loneliness compared with residents living in buffers with less than 10% green space. We re-analysed our cross-sectional data using a similar approach to [59] by examining the percentage of green space in 400, 800, and 1600 m but found no association between green space and loneliness in the middle to older adults living in Brisbane. While our present study found a similar null association between access to green space in the 400 and 800 m buffers and loneliness, the inconsistency of our result with [59]’s study in 1600 m buffers might be due to differences in the measurement of loneliness and our study’s specific age group [59]. In [59]’s study, they used a one-item question to measure loneliness, while this study used a three-item loneliness scale developed by authors in [121]. They in [121] used three question items: “How often do you feel left out, isolated from others, or lack companionship, “which is very different to the question “I often feel very lonely” used by authors in [59]. The stigma associated with loneliness may be another factor that may cause respondents to hesitate to admit loneliness and underreport their actual experiences [122]. Alternatively, direct questions on loneliness used by [59] might be rated differently from the experiences of loneliness measured by [122]. In a recent systematic review of 57 studies, objectively measured built environment factors showed no direct association with loneliness [61]. The authors did find, however, that it was people’s experience of the built environment that was associated with loneliness. Our study didn’t examine residents’ experience of using green spaces and focused instead on the access to quantity and quality green spaces, where people may or may not use these spaces. According to authors in [123], the disadvantaged population are less likely to use recreational facilities when available. That behaviour may be adopted for other outdoor activities. Indeed, future studies should look beyond simply addressing spatial access to the quantity and quality of green space to consider investigating how people use and experience green spaces.

Furthermore, authors in [120], in a cross-sectional study conducted in the Netherlands, found that loneliness in children, young, and older adults was lower when they had access to a higher percentage of green space within 3000 m of their home. They also found that access to green space within 1000 m of their home only contributed to lower loneliness in children and young adults but not in older adults. Our biggest buffer was 1600 m, while authors in [120] examined the larger 3000 m buffer. A larger distance buffer might be more effective in terms of the contribution to loneliness compared with smaller buffers. A meeting with someone important (of high quality) usually takes longer, and longer meetings may happen in a special place where distance is less important for location selection. A study shows that short meetings can occur closer to home, but longer meetings (with friends and family) are often organised further away from individuals’ homes [124]. Therefore, if social contact is with someone important, it could be that a green space further from homes is used (probably a location between individuals’ homes) and possibly that older people are happy to drive to green spaces rather than walk to their closest green space. Scholars also suggest that in everyday life, quality contact seems to be more limited [126–128]. Closer green spaces may be useful for a short meeting with neighbours and friends or sporadic encounters with neighbourhood contacts for whom the quality of social relationships might not be as strong as meeting with someone special like relatives, important friends and family. Furthermore, [125] found that people travel further distances to use a park that is perceived as attractive to them. In their study, prceived attractiveness appeared as a more important factor in using a park than proximity. It is possible that people may travel a long distance to find an attractive green space for gathering with their important friends or family members. Green space close to the home of middle to older adults that can be used regularly might be for transient spatial contact and interactions that are not strong enough to protect from loneliness. Future studies should also investigate if green spaces within a longer distance (longer than 1600 m) of older adults’ homes are more important than closer green spaces in terms of the impact on loneliness.

Finally, we hypothesised that access to high-quality and greater green space would contribute to neighbourhood inequity in loneliness. However, in our final model, we found that the differences in loneliness between disadvantaged and advantaged neighbourhoods were not explained by access to either quantity or quality of green space. This null-contribution was not unexpected since the initial model’s result showed that access to green spaces had no association with loneliness. This result is inconsistent with a study by authors in [120]. They observed that adults of low-income and low-educated groups experienced lower levels of loneliness in 1000 and 3000 m buffers with a higher percentage of green space. These inconsistent findings might be related to different locational contexts and measurements. For example, we used neighbourhood-level disadvantage, while authors in [120]’s study used individual disadvantage components. Future studies are needed that use the same measurements to allow comparison between studies to better understand the association between green space, disadvantaged neighbourhoods, and neighbourhood inequity in loneliness.

This study is of importance since the increasing population growth in urban areas [129], [130], combined with spatial densification strategies, has a severe negative impact on ecosystem services (e.g. green space reduction) [131], [132]. These changes can put people, especially the lower socio-economic status groups, at higher risk of being unhealthy because of increased urban heat, which has adverse ecological effects on health [133], [134]. Moreover, rapid urbanisation and population growth aggravate inequity by constraining government ability to deliver basic services and resources, limiting urban residents’ access to green spaces, particularly the low socio-economic status groups [135], who already live in less green neighbourhoods [136], [137]. These ongoing negative circumstances imply the need for evidence-based recommendations for future urban development, particularly in disadvantaged neighbourhoods, where residents are at greater risk of loneliness [23], [116], [138].

This study had several limitations but provided some useful suggestions for future research. Our study’s quality of green spaces was scored based on the green spaces dataset available in Brisbane City Council (BCC). We could not measure the quality of the other green spaces in Brisbane that were not governed and maintained by BCC, so they were removed. It is possible that informal green spaces (e.g., linear green spaces along railway lines) are also used by participants, and accurate access to comprehensive and consistently measured green space data is difficult to source in Australia. Therefore, future studies should go further by considering the quality of additional and possibly informal green spaces too. Furthermore, we measured the quality of green space based on datasets from the BCC, which were limited to specific green space characteristics. However, we did not have access to all characteristics of interest (e.g., safety and cleanliness) to calculate green space quality. Moreover, we had no understanding of the condition and usability of those facilities, such as being clean and safe. Moreover, we did not undertake any validation by the BCC data and cannot comment on its accuracy. However, as this dataset is maintained and updated biannually by BCC to “encourage third parties to develop apps, website and tools that can benefit Brisbane residents and businesses” [139], we anticipate few omissions. For a more accurate and actual understanding of the quality of green space, an in-depth investigation, including both objectively (e.g. site observation) and subjectively measured green space qualities, is needed [140]. The level of loneliness in the sample was generally low, and at least 50% of participants had a minimum score of 3 on a loneliness measure ranging from 3 to 9. It is possible, therefore, that the null associations were related to the small variance in loneliness. To further understand the contribution of green space to loneliness, more variance in loneliness might be needed, and additional research is required.

Our study was cross-sectional in design, which limits the interpretation of causality. Future research should consider investigating the impact of change in the quantity and quality of green space on a possible change in loneliness over time among middle to older adults of disadvantaged neighbourhoods. The current research sample included middle to older adults in Brisbane; different contexts in terms of population size, density, and socio-economic characteristics may yield different results. Consequently, our findings may not generalise to other age groups, populations, and settings (e.g., underdeveloped countries). Moreover, the 2007 HABITAT baseline sample was broadly representative of the Brisbane population aged 40–65 years at that time, although lower socioeconomic groups and residents of disadvantaged neighbourhoods were underrepresented [79]. The socioeconomic profile of the 2016 sample is similar to that observed in 2007 [141], hence loss-to-follow up over this period is unlikely to have seriously biased this study’s findings. However, the magnitude of the measured socioeconomic inequities in loneliness and greenspace in the 2016 sample are possibly smaller than the actual inequities in the population because of the socioeconomic underrepresentation.

Finally, although our loneliness measure is widely recognised and well-developed, quantifying loneliness remains a challenge. Loneliness is a stigmatised condition, and people may prefer not to admit to being lonely or interpret questions about social interactions differently to direct questions about loneliness. Despite these limitations, to the best of our knowledge, this is the first study to test the contribution of percentage and quality of green space in neighbourhood inequity in loneliness in a population of middle to older adults.

## Conclusion

Our study found that while the quantity and quality of green spaces were inferior in disadvantaged areas compared to advantaged neighbourhoods, none of the measures of green space was found to contribute to loneliness or the observed differences across neighbourhood disadvantage quintiles. Although green spaces can provide benefits such as opportunities for socialisation and physical activity, which can help combat loneliness, it appears that green spaces located within 1600 m of residents’ homes may not necessarily contribute to loneliness or neighbourhood inequities in loneliness. It is possible that residents may spend time with friends and family in green spaces that are farther away from their homes than 1600 m, thereby building and maintaining strong social relationships that can prevent loneliness. Our study suggests that the lack of association between green space and loneliness may be explained by the idea that strong, quality social relationships that prevent loneliness are not necessarily shaped and maintained by the availability and accessibility of green spaces close to where residents live, and that other green spaces farther away (more than 1600 m) from older individuals’ residences can have a greater influence on loneliness, which is needed to investigate in further studies. Additionally, it is important to understand that spatial residential proximity to green spaces does not necessarily mean that people will use or be exposed to them, which is important in gaining the benefits of green space. Therefore, future studies should consider the green spaces that residents use or are exposed to regularly in order to gain a more accurate understanding of the association between green spaces and loneliness. Furthermore, it would be beneficial to determine which types of green spaces (such as recreation, sport, national, corridor, or community parks), at what distance from residents’ homes, and with what levels of quality can contribute to a reduction in loneliness.

This study suggests that living in disadvantaged neighbourhoods may significantly contribute to loneliness later in life. However, further research is needed to identify the underlying mechanisms responsible for this association. Additionally, disadvantaged neighbourhoods with less green space and less access to high-quality green space can severely impact health and wellbeing. Urban and community planners should therefore consider an equitable distribution of green space, including facilities and amenities, when designing neighbourhoods. It is important to note that allocating new high-quality green spaces primarily in more advantaged neighbourhoods rather than disadvantaged areas could exacerbate health inequities. This research also supports neighbourhood-level assessments, interventions, and planning to reduce public health inequities associated with urban green space provision.

## Electronic supplementary material

Below is the link to the electronic supplementary material.

Supplementary Material 1

## Data Availability

The research data associated with the paper are available upon request from Gavin Turrell (gavin.turrell@rmit.edu.au).

## Footnotes

1 Park features included trails, paths, open spaces, swimming pools, playgrounds, sports fields, and skating areas. Park amenities are aspects that contribute to the attractiveness of a park. The observed park amenities included places to sit, ponds/lakes, drinking fountains, picnic areas, vending amenities, restrooms, tables, bike racks and parking lots.
