# Supplementary material for: Neighbourhood socio-economic disadvantage and loneliness: the contribution of green space quantity and quality
Source: BMC Public Health. 2023 Mar 30;23:598. doi: 10.1186/s12889-023-15433-0 (PMC10061840; doi:10.1186/s12889-023-15433-0)
Supplement: Supplementary file 1 — Supplementary Material 1 [file 12889_2023_15433_MOESM1_ESM.pdf]

Table 8: Interaction between quantity and quality of green space and loneliness by neighbourhood disadvantage, adjusting for the covariates[1]

	Green space 400 m buffer size	Green space 800 m buffer size	Green space 1600 m buffer size
Neighbourhood disadvantage (ref. Q5[2]) × Percentage of green space (ref. less than 10% green space percentage)			
Q1 × 10-20% Of green space	-0.007(-0.27, 0.26)	-0.136(-0.42, 0.15)	-0.217(-0.65, 0.21)
Q2 × 10-20% Of green space	0.093(-0.17, 0.36)	0.029(-0.28, 0.33)	-0.084(-0.58, 0.42)
Q3 × 10-20% Of green space	0.015(-0.22, 0.25)	-0.167(-0.43, 0.10)	-0.218(-0.73, 0.29)
Q4 × 10-20% Of green space	-0.060(-0.30, 0.17)	-0.082(-0.35, 0.18)	-0.058(-0.50, 0.39)
Q1 × 20-30% Of green space	0.138(-0.17, 0.45)	-0.307(-0.63, 0.02)	-0.281(-0.77, 0.21)
Q2 × 20-30% Of green space	-0.080(-0.37, 0.21)	0.083(-0.24, 0.41)	0.102(-0.41, 0.61)
Q3 × 20-30% Of green space	-0.122(-0.39, 0.15)	-0.174(-0.48, 0.13)	-0.147(-0.67, 0.37)
Q4 × 20-30% Of green space	0.026(-0.23, 0.28)	-0.239(-0.51, 0.04)	-0.034(-0.48, 0.42)
Q1 × >30% Of green space	0.007(-0.33, 0.34)	0.048(-0.36, 0.46)	-0.046(-0.57, 0.48)
Q2 × >30% Of green space	0.024(-0.29, 0.34)	0.220(-0.14, 0.57)	-0.090(-0.61, 0.43)
Q3 × >30% Of green space	-0.105(-0.39, 0.18)	-0.146(-0.47, 0.18)	-0.374(-0.93, 0.18)
Q4 × >30% Of green space	-0.242(-0.49, 0.01)	-0.112(-0.38, 0.16)	-0.179(-0.64, 0.28)
Neighbourhood disadvantage (ref. Q5) × Quality of green space (ref. lower quality)			
Q1 × Higher quality of green space	0.161(-0.05, 0.37)	0.030(-0.19, 0.25)	-0.175(-0.39, 0.04)
Q2 × Higher quality of green space	0.146(-0.06, 0.35)	-0.071(-0.28, 0.14)	-0.181(-0.39, 0.03)
Q3 × Higher quality of green space	-0.106(-0.29, 0.08)	-0.086(-0.27, 0.10)	-0.139(-0.33, 0.06)
Q4 × Higher quality of green space	-0.048(-0.23, 0.14)	-0.074(-0.26, 0.11)	-0.162(-0.35, 0.03)

1.Linear regression coefficients and their 95% confidence intervals
2.Q1 to Q5 are neighbourhood disadvantage quintiles
